# Intelligence

**Published:** 1833-01

**Authors:** 


### INTELLIGENCE.

King's College, London. The Medical School will open for the Spring Courses, on Monday, the 21st instant, in all the classes, excepting the Botanical, which will not commence till March.

Professor Lyell will begin a course of lectures on Geology in April.

Royal Institution. The evening meetings commence on Friday, the 25th instant, and the lectures towards the close of the month.

Professor Burnett will deliver a short and popular course of lectures on Botany here after Easter.

### Adjudication of Prizes for a Knowledge of Botany.

The Society of Apothecaries have laboured, zealously and wisely, in the improvement of medical education; and, by the well-judged exercise of the powers intrusted to them, the Courts of Assistants and Examiners have done more towards raising their department of the profession to the rank it deserves to hold, than could have been anticipated in so comparatively short a time.

To their patronage one branch of science is especially beholden; for, without their assistance, the means of its pursuit could scarcely have been within the reach of the London student: we mean Medical Botany. Convinced of the importance of the study, and perceiving the general want of materials for demonstration, they have most liberally opened the Chelsea Botanic Garden, which they have so long supported with so many sacrifices, and at such a great expense, to all medical students without exception; and they annually offer a gold and silver medal as a further encouragement for the prosecution of this study, far too generally neglected.

These medals were awarded on the 4th of December to Mr. Thomas H. Cooper, of the London University, and Mr. Philip K. Weston, of King's College.

We should have contented ourselves with the simple announcement of the fact, and honourable record of the names of the successful candidates, had not our attention been directed to the reports which have appeared in the public prints, some specimens of which we subjoin, and from these we learn that "this year was one of more excitement than usual," in consequence of the King's College students coming into the field for the *first time*, as competitors with those of the London University. As the College has only been open *one* year, and its alumni, of course, being at present tyros, we did not suppose that pupils of twelve month's standing would have caused so much excitement among the veterans of the elder sister; and we doubt not the Collegians are well pleased to find that, in their first attempt, made at the very first opportunity they have had, and after only one year's study, they have carried off the silver medal from a University student, of at least equal, if not longer standing, and made another of three or four times their date doubt whether he should gain the victory: indeed, we learn that the race was not won by much more than half a neck.

There is one part of the first of the following reports which seems obscurely worded, if not intentionally ambiguous. It entirely omits to mention that the silver medal was awarded to Mr. Weston: and it states that "Professor Lindley has the *entire superintendence* of the science of botany at the London University," which, with the plural mention of "professors" at the King's College, might lead to the supposition that the botanical professor there has not equally the *entire* superintendence of his department in that institution. Again, it is said that the College *Professors* were most sanguine in their hopes of sucecss. Now, we cannot suppose that reference is made to the professors of any other science, and yet there is but one professor of botany in the College; and he, we are assured on unexceptionable testimony, never expressed any such sanguine hopes. We know he stated that there were some students in his class who would compete with every probability of success with any pupils of *equal* standing: this opinion he expressed before the examination, and we know he retains it still: indeed, the results of the competition has proved it to be correct; for Mr. Cooper was not the only candidate that the University sent up; and, were it wanted, the one series of answers which were wholly, and the other which were partly read, might be given as further proof.

*From the Morning Chronicle.*

"The London University has recently gained an additional laurel in having awarded to one of its students in botany, (Mr. Cooper,) after a most minute and strict examination at Apothecaries' Hall, the gold medal. The competitors for this grand prize were the students of King's College, the professors of which were most sanguine in their hopes of success. Professor Lindley has the entire superintendence of the science of botany at the London University."

*From the Lancet.*

"On Tuesday the annual medals for botany were thus distributed to the successful candidates by the Society of Apothecaries:

"The gold medal, to Mr. Thos. H. Cooper, of Lewes, Sussex, student of the London University.

"The silver medal, to Mr. Philip King Weston,* of Boxhill, Surrey,† student of King's College.

"The gold medal had on one side the head of Linnæus, and upon the other the figure of a youth (possibly emblematical of the successful candidate,) lying at the feet of Flora, who is recommending to his notice, Ray, Linnæus, Jussieu, and Sloane, whose names are engraved upon a monument by her side. The whole is well executed; its nominal value is £10., and is certainly worth, at least, that sum: it is by Wyon, of the Mint. The silver one is from the same die. The giving of these medals is one of the very few acts, if not the only one, which the Apothecaries' Company contributes to promote the zeal of medical students; and this causes much emulation among them. This year was one of more excitement than usual, in consequence of the examination being the first at which the King's College students have attempted to contest the gold medal with those of the London University, who have carried it off each year it has hitherto been given; this making the third time.‡

"The answers to the questions given by Mr. Cooper, the successful candidate, were in the highest degree creditable to that gentleman.§ The manner, also, in which Mr. Griffiths|| and Mr. Valentine distinguished themselves in displaying their knowledge of vegetable anatomy was highly gratifying to all parties. Indeed, the gentlemen of the London University are, we understand, all of them good botanists, and the King's College men will have hard work to beat their contemporary students, and be no mean botanists either."

*From a Correspondent in the Medical Gazette.*

"The gold and silver medals annually offered by the Society of Apothecaries to all students (not being apprenticed to members of the Society,) were, on Tuesday the 4th instant, awarded to Mr. T. H. Cooper, of the London University, and Mr. P. K. Weston, of King's College.

* "Mr. Weston obtained the first prize at King's College last year.

† An error, for Hare Hall, Essex.

‡ This we believe to be an error; for, if our memory does not betray us, it was a student of the London *Hospital*, not of the London University, who gained the prize the first year.

§ "There were forty-four questions, for the replies to which three hours were given.

|| "Mr. Griffiths obtained the gold medal last year."

"As these prizes are given, not merely for comparative, but for absolute merit and positive proficiency, (last year only one medal was decreed, the other being withheld,) it is highly creditable to these young men, and gratifying to their friends, to have such evidence of the right employment of their time, and to be thus honourably distinguished as the two best botanists that the metropolitan schools this year afford.

"Long before the examination took place, we heard that Messrs. Cooper and Weston were the favourite candidates; and from the former gentleman being a student of several years standing, and the latter of one year only, it was generally expected that the prizes would be awarded as they have been. We therefore think it unwise, and we are sure it must be contrary to his wishes, that the success of the gold medallist should be mentioned by his would-be friends as a triumph over his fellow candidate, to whom the silver medal was decreed. At any time, we should have thought such reports ungenerous: under present circumstances we think the exultation foolish; for, although the success of the senior student is highly honourable, it can scarcely be regarded as a triumph over a competitor so much his junior; and our veracious contemporary, when affirming that this was 'the first examination at which the King's College students have attempted to contest the gold medal with those of the London University,' would have done well to have recollected that the College was only opened in October 1831, so that it was the very first opportunity of competition which has occurred."

---

Mr. Alfred Swaine Taylor has been appointed the successor of the late Mr. Alexander Barry, as joint lecturer on Chemistry with Mr. Aikin, in the medical school of Guy's Hospital.

---

At a meeting of the Medico-Botanical Society of London, on the 11th ult., Sir Anthony Carlisle, a member of the Society, communicated to the meeting that he had just received a small quantity of the expressed juice of a succulent tree, of the genus Cactus, from the province of Senora, in the Gulph of California, which, it is asserted, upon the authority of three respectable gentlemen, actually cures Hydrophobia when this disease has taken effect. He (Sir A. C.) therefore conceived it of the highest importance that this reputed remedy should be made known to the medical profession and the public through this Society, in order to procure it a fair trial in the first case which might present itself: in furtherance of which object, the learned gentleman stated to the Society that he would gladly accompany any professional gentleman who might have the care of a case of hydrophobia, either in private or hospital practice, in order that he might report the effects of this remedy, and direct the administration of it according to the rules given to him.

**Dec. 6.**

Henry Bowen, St. Albans
Henry Stanhope Illingworth
William Salter, Whitchurch, Herefordshire
Robert Stopford Taylor, Sheffield

**Dec. 13.**

Alexandor Angus, Greek street, Soho
John Crea, Whittingham
Robert Davis, George street, New road
Henry Goddard
Alfred Higgenson, Derby
James Henly Hicks, Plymouth

**Dec. 20.**

Charles John Brickwell, Banbury
George Alfred Davenport, Market Harboro'
John Enscoe
John Hunt, Thumby, Leicestershire
Charles Potter, Brockhurst, Hants

**Dec. 28.**

John Carter, Coventry
William Hall, Leatherhead.

### METEOROLOGICAL REGISTER,

**By Messrs. Harris and Co. Mathematical Instrument Makers, 50, High Holborn.**

| | Moon. | Rain gauge. | Thermometer. | | | Barometer. | | De Luc's Hygrometer | | Winds. | | Atmospheric Variation. | | |
|---|---|---|---|---|---|---|---|---|---|---|---|---|---|---|
| Nov. | | | 9a.m. | max. | min. | 9a.m. | 10p.m. | 9a.m. | 10p.m. | 9 a.m. | 10p.m | 9 a.m. | 2 p.m. | 10 p.m. |
| 20 | | | 48 | 53 | 48 | 29.51 | 29.48 | 79 | 79 | SE | SE | cloudy | fine | fine |
| 21 | | | 51 | 54 | 42 | .40 | .40 | 78 | 79 | ESE | ESE | foggy | | foggy |
| 22 | ● | | 47 | 51 | 42 | .57 | .69 | 76 | 75 | SE | SE | | | |
| 23 | | | 48 | 54 | 48 | .74 | .71 | 77 | 75 | SE | S | | | |
| 24 | | | 52 | 56 | 44 | .72 | .71 | 75 | 75 | S | S | | | fine |
| 25 | | | 48 | 53 | 45 | .57 | .39 | 78 | 78 | SW | SW | rain | cloudy | cloudy |
| 26 | | .37 | 47 | 48 | 41 | .36 | .49 | 75 | 75 | WSW | W | foggy | rain | fine |
| 27 | | | 44 | 47 | 41 | .51 | .30 | 75 | 77 | W | W | fine | fine | foggy |
| 28 | | .08 | 43 | 50 | 42 | .46 | .62 | 75 | 76 | W | SW | fine | cloudy | rain |
| 29 | | .28 | 44 | 46 | 38 | .42 | .36 | 74 | 73 | WSW | WSW | foggy | fine | cloudy |
| 30 | ☽ | | 42 | 54 | 43 | .66 | .53 | 72 | 75 | W | S | | rain | cloudy |
| Dec. | | | | | | | | | | | | | | |
| 1 | | .18 | 54 | 56 | 44 | .60 | .60 | 77 | 78 | W | WSW | | cloudy | fine |
| 2 | | | 49 | 56 | 44 | .38 | .32 | 75 | 77 | SW | WNW | rain | fine | cloudy |
| 3 | | .12 | 45 | 48 | 43 | .32 | .43 | 70 | 70 | WNW | NW | fine | rain | fine |
| 4 | | | 45 | 52 | 41 | .74 | .92 | 69 | 73 | N | N | | fine | rain |
| 5 | | .03 | 42 | 45 | 40 | 30.01 | .94 | 72 | 74 | N | WNW | foggy | | rain |
| 6 | | .14 | 44 | 46 | 42 | 29.83 | .91 | 75 | 75 | N | NNE | sleet | fine | fine |
| 7 | ○ | | 43 | 46 | 40 | 30.07 | 30.14 | 76 | 76 | NNE | SSE | foggy | fine | cloudy |
| 8 | | | 43 | 48 | 42 | .14 | .15 | 78 | 79 | SW | WSW | fog | | |
| 9 | | | 45 | 49 | 46 | .15 | .15 | 81 | 82 | WSW | WSW | | cloudy | foggy |
| 10 | | .03 | 48 | 50 | 42 | .11 | .13 | 81 | 81 | SW | SW | foggy | cloudy | fine |
| 11 | | | 45 | 49 | 45 | .18 | .22 | 79 | 79 | SW | W | | fine | fine |
| 12 | | | 48 | 51 | 42 | .23 | .20 | 78 | 76 | WNW | WNW | foggy | cloudy | foggy |
| 13 | | | 45 | 49 | 41 | .01 | 29.88 | 76 | 75 | W | SSW | | | |
| 14 | ☾ | | 46 | 52 | 43 | 29.75 | .72 | 75 | 76 | SW | SW | | fine | cloudy |
| 15 | | | 51 | 53 | 36 | .35 | .68 | 79 | 77 | SW | NW | rain | rain | fine |
| 16 | | | 45 | 54 | 47 | .56 | .42 | 79 | 81 | SW | SW | foggy | fine | rain |
| 17 | | .12 | 52 | 55 | 42 | .36 | .30 | 80 | 78 | WSW | SW | foggy | cloudy | rain |
| 18 | | | 45 | 51 | 37 | .36 | .46 | 74 | 72 | W | W | fine | fine | fine |
| 19 | | | 39 | 42 | 35 | .51 | .51 | 73 | 75 | W | W | foggy | | cloudy |

The quantity of Rain fallen in November, was 1 inch and 89.100ths.

